# A Python Code for Simulating Single Tactile Receptors and the Spiking Responses of Their Afferents

**DOI:** 10.3389/fninf.2019.00027

**Published:** 2019-04-17

**Authors:** Qiangqiang Ouyang, Juan Wu, Zhiyu Shao, Miao Wu, Zhiyong Cao

**Affiliations:** State Key Laboratory of Bioelectronics, School of Instrument Science and Engineering, Southeast University, Nanjing, China

**Keywords:** tactile receptor, tactile afferent, electromechanical circuit, two-channel filter, spike synthesizer, code: python

## Abstract

This work presents a pieces of Python code to rapidly simulate the spiking responses of large numbers of single cutaneous tactile afferents with millisecond precision. To simulate the spike responses of all the major types of cutaneous tactile afferents, we proposed an electromechanical circuit model, in which a two-channel filter was developed to characterize the mechanical selectivity of tactile receptors, and a spike synthesizer was designed to recreate the action potentials evoked in afferents. The parameters of this model were fitted using previous neurophysiological datasets. Several simulation examples were presented in this paper to reproduce action potentials, sensory adaptation, frequency characteristics and spiking timing for each afferent type. The results indicated that the simulated responses matched previous neurophysiological recordings well. The model allows for a real-time reproduction of the spiking responses of about 4,000 tactile units with a timing precision of <6 ms. The current work provides a valuable guidance to designing highly realistic tactile interfaces such as neuroprosthesis and haptic devices

## Introduction

Tactile receptors are sensory receptors that respond to mechanical pressure or distortion by producing action potentials (spikes) in their associated afferents (Zhu and Rozell, 2015). Previous neurophysiological research has found that four types of tactile afferents in skin are responsible for tactile sensation (Vallbo and Hagbarth, 1968; Johnson, 2001). Different types of tactile afferents respond to mechanical stimuli with different selectivity. Slowly adapting type 1 (SA1) afferents respond to sustained pressure and low-frequency vibrations and convey information about shape (Goodwin et al., 1997). Rapidly adapting type 1 (RA1) afferents respond to stimuli that bump against the skin and convey information about motion across the skin (Johansson and Westling, 1987). The RA1 and SA1 afferents account for spatial acuity in psychophysical tests (Johansson and Vallbo, 1979). Rapidly adapting type 2 (RA2) afferents, also called Pacinian Corpuscle (PC) afferents, are very sensitive to high frequency vibrations and are thought to convey information about distal events such as texture roughness sensed through a tool (Yoshioka et al., 2007). Slowly adapting type 2 (SA2) afferents are sensitive to skin stretch and may provide information about the hand conformation and posture during grasping and other hand movements (Collins et al., 2007). The SA2 afferents have never been observed in neurophysiological studies of mechanoreceptors in the monkey hand (Johnson, 2001). While the response properties of three major types of tactile afferents (SA1, RA1 and PC) have been extensively studied in neurophysiological recordings. Therefore, a model of reproducing responses of SA1, RA1 and PC afferents is feasible, and valuable to investigating tactile sensation.

Generally a model of simulating cutaneous afferent response should at least consist of the receptor model to emulate the selectivity of each afferent type, and the spiking neuron model to generate action potentials in afferents. In current work we treat the tactile receptor and its associated afferents as a single tactile unit. A number of influential models have been developed to characterize how a spiking neuron produce action potentials. The most important and accurate of the early neural models is the Hodgkin–Huxley model (Hodgkin and Huxley, 1952), which describes a spiking neuron by a coupled set of four ordinary differential equations (ODEs). The Hodgkin-Huxley model has inspired several more simplified models, such as the Morris-Lecar model, FitzHugh-Nagumo model and Izhikevich model, all of which have two coupled ODEs (Fitzhugh, 1961; Izhikevich, 2003; Tsumoto et al., 2006). The integrate-and-fire (IF) model is another spiking neuron model that has computational simplicity, scalability and applicability to simulate SA1, RA1, and PC afferents (Bensmaia et al., 2008; Becker et al., 2009; Saal et al., 2017).

The previous work of spiking neuron has been fruitful. Besides in neuromorphic applications, some models of mechanotransduction have also been reported to characterize the tactile afferents by using above spiking neuron models (Spigler et al., 2012; Rongala et al., 2015; Nguyen et al., 2018; Osborn et al., 2018). However without considering the filter characteristics of tactile receptor, the reproducing of selective responses (e.g., frequency and adaption) of the three major types of tactile afferents is not mentioned in these work. By combining a filter model of tactile receptor with IF neuron model, some researchers developed more comprehensive models that can replicate many response properties of SA1, RA1, and PC afferents and even the afferent population responses (Dong et al., 2013; Saal et al., 2017). However, most previous spiking neuron models including the IF model fail to accurately recreate the neural spiking responses of tactile afferents, particularly for the PC afferents (Biswas et al., 2015).

Accurate characterization of the evoked action potentials in response to a stimulus is paramount to designing neuroprostheses with biological compatibility (Marmarelis, 1993; Bertaccini and Fanelli, 2009). In tactile neural nerve system, the spike timing is important to encoding object properties including vibratory frequency, surface texture, and surface curvature (Saal et al., 2016). Recent literature hints that the binary spike trains of the sensory receptors can be quite accurately modeled with an pulse frequency modulator (PFM) (Biswas et al., 2015). The non-linear stochastic mechanotransduction (NSM) model consisting of a mechanic filter and an adaptive relaxation PFM allows for accurate reproduction of the spiking response of PC afferent and captures the shape of the action potential more accurately than the IF model (Biswas et al., 2015). Hower the NSM model which has more than 10 coupled ODEs can only be implemented to recreate the responses of PC afferents, and is too complicated to allow real-time simulation of massive numbers of tactile units.

The goal of current work was to propose an model that could accurately recreate most single afferent response properties observed in previous literature, but that was simple enough to allow for fast computation. Most previous models of recreating afferent response include a model of skin mechanics. However, previous studies have shown that incorporating implementing skin mechanics is not necessary to achieve high spiking precision for only reproducing responses of the tactile afferents located under stimuli sites (Saal et al., 2017), and previous work was able to reproduce precise spiking patterns evoked by vibrating probes without implementing a skin mechanics model (Kim et al., 2010; Dong et al., 2013). The model of skin mechanics can be seen as a model of computing the indentation distribution over the skin from the pressure distribution. There are already much experimental data about tactile afferent responses to skin indentation depth (not applied force) in neurophysiological literature [e.g., Muniak et al. (2007)]. Under a certain area of surface indenting the skin, the caused indentation depth on stimulus site change linear with applied pressure according to elastic theory (Timoshenko and Goodier, 1970). In order compare simulated responses with well-established response properties in previous neurophysiological literature, the current work only focus on simulating afferent responses of single unit to skin indentation, which is the basic work for recreating afferent population responses.

A cutaneous tactile unit involves not only the filtering of the mechanical signal but also the modulation of the electrical signal (Biswas et al., 2015). Inspired by previous filter models of tactile receptor and frequency modulator of generating spikes, we proposed an electromechanical circuit model consisting of a two-channel filter (TCF) to characterize the mechanical selectivity of a single tactile receptor and a spike synthesizer using frequency modulator to accurately recreate action potentials evoked in afferents. In the TCF model, the signal of skin indentation is selected by the two-channel filter, in which a low-pass filter (LPF) and band-pass filter (BPF) were designed to select the static and dynamic components of the response, respectively. The spike synthesizer was designed to convert the the output signal of TCF model into action potentials.

The main contributions of this work are as follows: (1) it presents a electromechanical circuit model that allows a rapid simulation of thousands of SA1, RA1, or PC afferents in response to skin indentation; (2) it presents a spike synthesizer to rapidly generate action potentials with millsecond timing precsion, which may improves the biological compatibility for neural prostheses; (3) it presents a method to train the parameters of the TCF model using neurophysiological firing-rate datasets and yield spike timing precision of <6 ms.

## Methods

**Figure 1:**
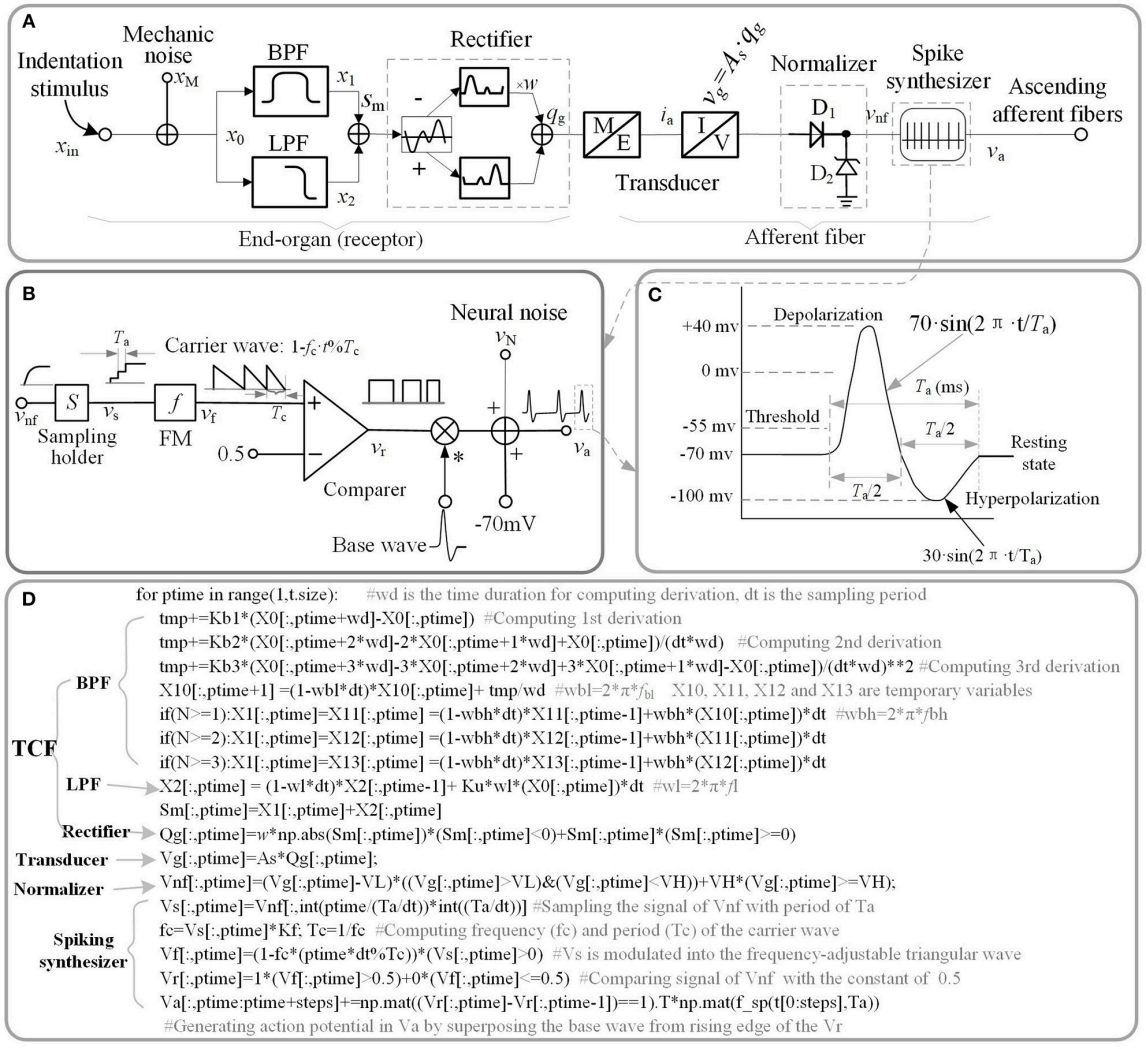
Schematic diagram of current model. **(A)** Overall model of single tactile unit. *x* stand for the indentation displacement produced in the skin. **(B)** Schematic diagram of spike synthesizer. **(C)** Typical shape of a spike. **(D)** Source code of implementing schematic diagram. The ‘f_sp()' is the function of the waveform shown in **(C)**.

The electromechanical model as illustrated in Figure 1A was developed to replicate the afferent response properties of single tactile unit. The whole model is comprised of the TCF model to characterize mechanoreceptive end-organ (the receptor) and other models (Transducer, Normalizer, and Spiking synthesizer) to characterize afferent fiber (the nerve).

### Electromechanical Circuit Model

A good way to investigate the response properties of single tactile unit is isolating from the afferent population (Muniak et al., 2007). Previous neurophysiological experiments of single tactile unit has indicated that stimulus intensity is encoded in the firing rate (ips, impulses per second) of action potentials rather than its firing amplitude (Goodwin et al., 1997; Muniak et al., 2007). In this work, we proposed the TCF model to quantitatively replicate the firing-rate response (*v*_nf_) of a tactile unit to mechanical indentation, then constructed a spike synthesizer to convert the signal of *v*_nf_ to corresponding to spikes. The main source code that implements the proposed model is depicted in Figure 1D, which are available in the link: https://github.com/ouyangqq/model_of_single_tactile_unit/blob/master/Receptors.py. In this code, all the intermediate and I/O signals were defined as two-dimensional Numpy arrays (spatial^*^temporal) to support simulating afferent population responses in future. To speed up computation, all logistic and loop operations were converted into vector or matrix operations using Numpy library imported as “np” in the source code.

#### Model of Receptor

Previous neurophysiological experiments indicated that each type of single tactile unit appears to mediate specific portions of the overall stimulus frequency characteristic (Bolanowski et al., 1988). SA1 afferents are most sensitive between 0.3 and 3 Hz (Johnson et al., 2000), RA1 afferents between 2 Hz and 50 Hz, and PC afferents between 30 and 500 Hz (Mountcastle et al., 1972). These characteristics of neural threshold are close to characteristics of a filter to the mechanical stimulus (skin indentation). Thus, we designed a two-channel filter to select the mechanical stimulus in frequency range of sensitivity for each type of tactile units. The Laplace transfer function of the filter is given as below.

(1) $$H_{\left(s\right)}=\;\frac{S_{m}(S)}{x_{in}(S)}=\;\underset{BPF}{\underset{︸}{\frac{K_{b1}\cdot S+K_{b2}\cdot S^{2}+\ldots +K_{b}n\cdot S^{n}}{S+2\pi \cdot fBL}\cdot {\left(\frac{2\pi \;\cdot fBH}{S+2\pi \cdot fBH}\right)}^{n+1}}}+\underset{LPF}{\underset{︸}{\frac{Ku\cdot 2\pi \cdot fL}{S+2\pi \cdot fL}}}$$

Where *K*_u_ and *K*_b(1~*n*)_ are weights of the low-pass filter (LPF) and the band-pass filter (BPF), respectively. *n* is the highest order of taking derivation to the input stimulus. *f*_*B*__L_ and *f*_*B*__H_ are the lower limit and upper limit cut-off frequencies of the BPF, respectively. *f*_L_ is the cut-off frequency of the LPF.

As illustrated in Figure 1A, the outputs of the two filters are added together. *x*_in_, *s*_m_ in equation (1) is the input stimulus and the added signal of the two filters, respectively. For convenience of processing, the added signal was rectified to a positive signal. *w* is the negative contribution for rectifying. *w* = 0 for SA1 afferents, since they tend not to respond to the offset of stimuli (Mountcastle et al., 1966); *w* > 0 for RA1 and PC afferents. *A*_s_ is the mechanotransduction coefficient. The transfer function *H*_(S)_ was converted to a differential equation, which can be discreetly solved in loop iterations as shown in Figure 1D. In the TCF model, the LPF mainly cares about the static component of the input stimulus, while the BPF is only sensitive to changes of stimulus (the dynamic component). According to previous work, the SA1 afferent is not only sensitive to static but also dynamic stimuli (Johnson, 2001). SA1 afferents respond approximately ten times more during dynamic stimulation than during static stimulation (Johnson et al., 2000). *K*_u_ was set to zero For the RA1 and PC afferents, since they adapt so quickly and tend not to respond during static stimulation (Johnson, 2001). As illustrated in equation (1), the current model includes several inertia components (1/(S+2π · *f*)), which causes different time delay between mechanical deformation on the skin surface and mechanical deformation of the receptor for each type of tactile units.

#### Model of Afferent Fiber

The transducer in Figure 1A proportionately converts the mechanical signal to electric current (*A*_s_ proportion coefficient of transducer), and drives the subsequent circuit to evoke spike trains in the ascending afferents.

The tactile afferents tend not to fire until the stimulus amplitude exceeds a minimum amplitude (the lower threshold) (Vallbo and Johansson, 1984), and the firing rate of tactile afferents do not increase when the stimulus amplitude exceeds a maximum amplitude (the upper threshold) (Freeman and Johnson, 1982). To simulate this neural threshold property similar with a normalizer, a rectifying diode D_1_ of lower limit, and a Zener diode D_2_ of upper limit were added in the model. Turn-on voltage (*V*_L_) of D_1_ was set as the lower limit of the action potential relative to the resting state (Figure 1C). For a typical neuron, the resting potential is around −70 millivolts (mV) and the threshold potential is around −55 mV. Thus, in the current study, *V*_L_ was set to 15 mV. The breakdown voltage (*V*_H_) of D_2_ was set to 1,000 mV.

The frequency response characteristics of the filter can obtained by substituting S = *j*·2π·*f* into equation (1). The frequency response characteristics of the neural threshold (*u*m) is given as follows:

(2) $$\begin{array}{r} T_{\left(f\right)}=\frac{V_{L}^{\;}}{A_{s}\cdot |H\left(j\cdot 2\pi \cdot f\right)|\;} \end{array}$$

Where *j* is the imaginary unit, *f* is the frequency. The lowest indentation threshold (*T*_low_) of a tactile unit can be evaluated as following equation.

(3) $$T_{low}=T_{\left(\frac{f_{BL}+f_{BH}}{2}\right)}=\frac{V_{L}^{\;}}{A_{s}\cdot |H\left(j\cdot 2\pi \cdot (\frac{f_{BL}+f_{BH}}{2})\right)|\;}$$

The spike synthesizer as illustrated in Figure 1B was developed to produce a biological neural spike with a firing rate that is proportional the signal of *v*_nf_. *v*_nf_ is the output of the neural threshold model. The *v*_nf_ was sampled (sampling period: *T*_a_) with holding into *v*_s_, and the *v*_s_ was then modulated into the frequency-adjustable triangular wave (*v*_f_). The *v*_f_ was then compared with a constant (0.5) to produce a pulse wave (*v*_r_). The *K*_f_ in Figure 1B was defined as the max firing-rate for each afferent tpye. *f*_c_ (1/*T*_c_ = *K*_f_·*v*_s_) is the frequency of carrier wave. The action potentials (*v*_a_) were finally generated by superposing the base wave from rising edge of the pulse wave. The base wave was synthesized with a typical spike shape as shown in Figure 1C. *T*_a_ is the period of a spike, which was set to 4 ms for each afferent type.

#### Model of Noise

As shown in Figure 1A, the mechanical and neural noises are separately incorporated in current model. The reason behind these two separate provisions is that the non-linear signal processing in tactile receptor and afferent fibers alters the statistical and morphological properties of mechanical noise (*x*_*N*_) and neural noise (*v*_*N*_) differently (Muniak et al., 2007). For simplicity the mechanical noise was considered as pseudo-Gaussian noise with 0.1 um of standard deviation and filtered with 1,000 Hz cut-off first-order low pass filter (Biswas et al., 2015). Neural noise was modeled as an additive random noise associating with the action potential (*v*_a_ see Figure 1C). For simplicity the neural noise was considered as pseudo-Gaussian noise with SD = 10 mv and filtered with 1,000 Hz cut-off first-order low pass filter.

### Model Training

The neurophysiological firing-rate datasets (recorded from rhesus macaques, see **Figure 3** in Muniak et al., 2007) in response to sinusoidal vibrations, were used as the training data. Before training, the firing-rate data was normalized using a feature scaling method to bring all values into the range of 0 to 1. The normalized firing rate of this model in response to peak amplitude (*x*) of sinusoidal stimulus and frequency (*f*) is illustrated in the following equation.

(4) $$\begin{array}{r} h_{\theta }\left(x,f\right)=A_{s}\cdot |H\left(j\cdot 2\pi \cdot f\right)\cdot x\;|\cdot \frac{1+w}{\pi } \end{array}$$

Using the above equation, the loss function of training the model was obtained as follows.

(5) $$J_{\left(\theta \right)}=\frac{1}{2}\cdot \sum_{i=0}^{m}{\left(h_{\theta }\left(x^{\left(i\right)},f^{\left(i\right)}\right)-y^{\left(i\right)}\right)}^{2}$$

Where *m* is the size of the training dataset, *y*^(i)^ is the target value of *i*_th_ training set. θ = [*K*_b1_, *K*_b2_, …, *K*_bn_, *K*_u_, *f*_BL_, *f*_BH_, *f*_L_, A_s_, *w*] is the parameter array of the model.

The goal of optimizing the parameters of the model is to find values of θ so that *J*_(θ)_ reaches its minimum. There are three major parameters optimization methods: gradient descent (steepest descent), Newton's method and the quasi-Newton method. The quasi-Newton method is good enough to produce superlinear convergence to a global minimum, thus the improvement over the steepest descent is dramatic especially on difficult problems (Wright and Nocedal, 2006). Since second derivatives are not required, the quasi-Newton method is more efficient than Newton's method (Wright and Nocedal, 2006). We implemented the BFGS quasi-Newton method to train the parameters using Scipy.optimize library (https://github.com/scipy/scipy/blob/v1.1.0/scipy). During updating, *K*_u_ and *f*_L_ were constrained to 0 for RA1 and PC units. *w* is constrained to 0 for SA1. The learning stepsize (α) was set to 0.55. The partial derivative of *J*_(θ)_ to each entry of θ was solved using central-difference formula. All the parameters were initialized to zeros. To evaluate the parameter variation as shown in Table 1, the whole training was repeated for 10 times.

**Table 1:** Summary of parameters (mean ± SD).

**Parameters**	**Mean in this model**	**SA1**	**RA1**	**PC**
*K_*u*_*	Weight of LPF	0.094 ± 0.031	0	0
*K*_b1_	Weight of 1st order BPF	0.205 ± 0.008	0.232 ± 0.021	0
*K*_b2_	Weight of 2rd order BPF	0	0.0031 ± 0.00021	0.128 ± 0.014
*K*_b3_	Weight of 3th order BPF	0	0	0.00111 ± 0.00011
*f_*BL*_*	Lower cutoff Frequency of BPF (Hz)	8.01 ± 0.21	60.10 ± 1.61	80.40 ± 1.82
*f_*BH*_*	Upper cutoff Frequency of BPF (Hz)	10.03 ± 0.41	80.09 ± 2.32	220.02 ± 4.21
*f_*L*_*	Upper cutoff Frequency of LPF (Hz)	100.20 ± 0.72	0	0
*A*_s_	Coefficient of transducer (V/mm)	3.80 ± 0.13	44.00 ± 1.33	0.36 ± 0.03
*w*	Negative contribution for rectifying	0	0.015 ± 0.002	0.212 ± 0.021
*K*_f_	Firing-rate encoding coefficient	180	200	300

**Figure 2:**
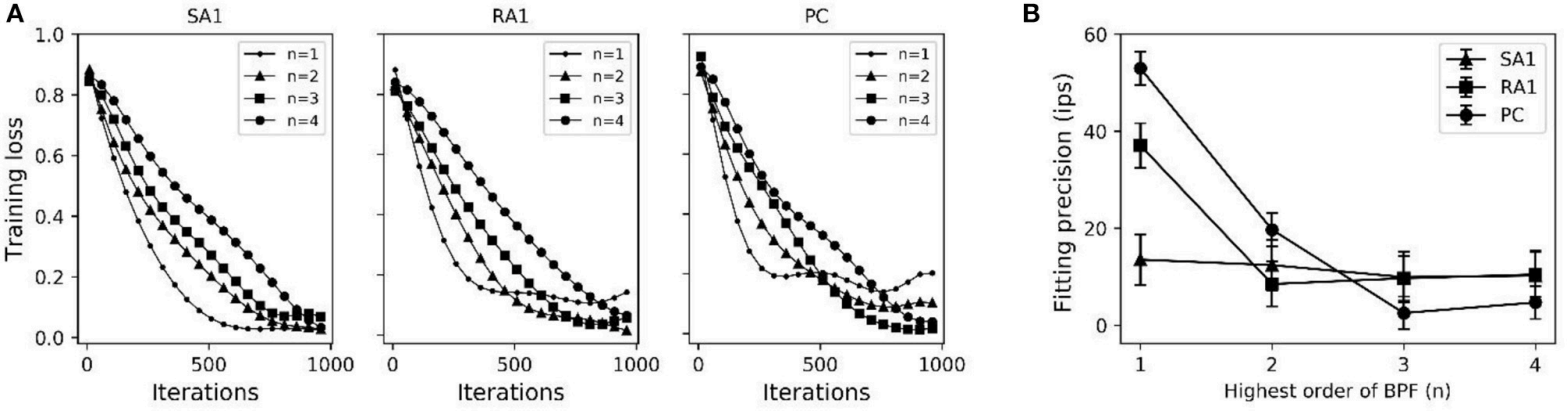
**(A)** Averaged loss value (*J*_(θ)_) changing with iterations under different highest order of BPF (n). **(B)** Fitting precision changes with highest order of BPF. Error bars represent the standard deviation. The difference between the fitting precisions at two orders was evaluated using *post-hoc* tests.

As seen in Figure 2A, after training of about 1000 iterations, the model for each type of tactile unit can be trained to be convergent (i.e., *J*_(θ)_ remains stable). The fitting precision in Figure 2B represents root mean squared error between the predicted firing rates and observed ones. The fitting precision was calculated by taking mean square root of *J*_(θ)_/*m* across from the 1000th to the 1100th iteration, then multiplying with *K*_f_. The *K*_f_ was set to 180, 200 and 300 for SA1, RA1, and PC units, respectively, referring the maximum firing rate of each afferent type (Muniak et al., 2007). The fitting precision changing with the highest order of BPF is shown in Figure 2B. The fitting precision decreased significantly from order 1 to 3 for the PC units (*P* = 0.028, ANOVA), and from order 1 to 2 for the RA1 units (*P* = 0.022). While there was no significant decrease with the rise of order for the SA1 units (*P* = 0.486). For a trade-off between precision and fast computation, the highest orders for SA1, RA1 and PC receptor was set to 1, 2, and 3, respectively. The summary of parameters obtained by this training method at the preferred set of highest order of BPF are shown in Table 1.

## Examples of Implementing the Model to Simulate Tactile Afferent Responses of Single Units

In the following section, we systematically compare simulated responses of tactile units with published data across well-established response properties in previous literature. We simulated the responses of single tactile unit to stimulus scaled as indentation depth. Simulations were performed on a personal computer with Intel processor i7-7700HQ, 8 GB of memory. The source code of current model and all simulations was written in Python3.6 using Numpy libraries and has been uploaded on a Github repository (https://github.com/ouyangqq/model_of_single_tactile_unit). In all simulations, we used parameters given in Table 1.

### Reproducing Action Potentials Above Threshold

**Figure 3:**
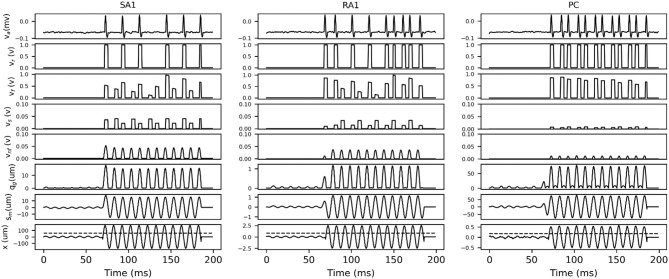
Details of intermediate signals in response (100 ms frame) to two typical sinusoidal stimuli with frequency of 100 Hz and peak amplitude below (first 37 ms) and above (37 to 100 ms) the threshold for afferent type. The black dotted line in bottom trace indicates the threshold at 100 Hz, which was calculated according to equation (2) by substituting the fitting parameters in Table 1. *x*, skin mechanical indentation; *s*_m_, mechanical output of TCF; *q*_m_, mechanical output of rectifier; *v*_nf_, voltage encoding normalized firing rate; *v*_s_, voltage of sampling firing rate; *v*_f_, voltage of frequency modulation; *v*_r_, comparer output voltage; *v*_a_, Action potential.

In order to describe how the TCF and spike synthesizer covert mechanical indentation (*x*_in_) into the action potential (*v*_a_), the details of intermediate signals in response to typical sinusoidal stimuli are presented. As shown in Figure 3, each type of tactile unit will not evoke spike activity until the amplitude of stimulus exceed its threshold. The evoked spike captures the typical shape of action potential as shown in Figure 1C.

### Reproducing Selective Responses of Tactile Afferents

The adaptation and frequency sensitivity are two basic selective response properties of a tactile unit. Hence a simulated experiment was conducted to evaluate the effectiveness of current model in predicting the selective responses recorded in previous neurophysiological experiments. A animation of simulating the selective responses of single tactile units can be found in Supplementary Material.

#### Tactile Sensory Adaptation

**Figure 4:**
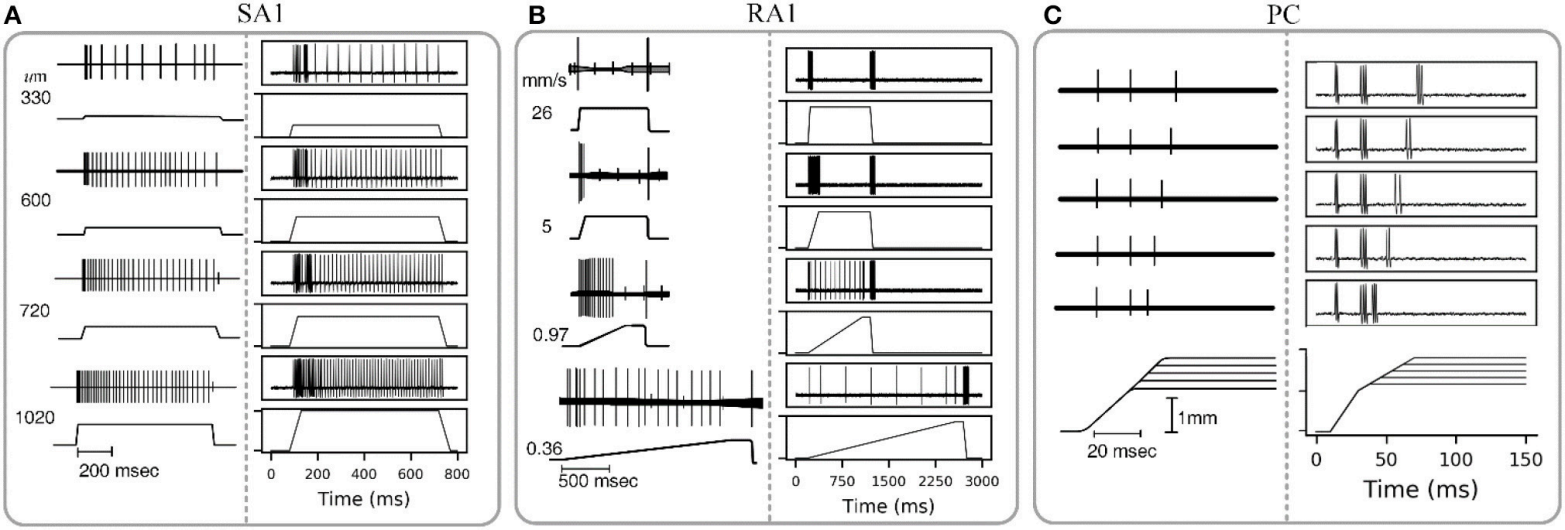
Adaptation properties of different types of tactile units. The recorded data of SA1, RA1, and PC are adapted from Mountcastle et al. (1966), Talbot et al. (1968), and Knibestöl (1973), respectively. **(A,B)** Recorded (left) and simulated (right) responses of single SA1 unit to ramp-and-hold indentations at different indentation depths, and single RA1 unit to ramps approaching indention of 850 um at different speeds. The stimulus amplitude and time course are shown in the lower trace of each pair; the upper trace shows the action potentials recorded from the sensory nerve fiber in response to the stimulus. **(C)** Recorded (left) and simulated (right) responses of a PC unit to stimulus with reduction of indentation amplitude. The upper traces show the recorded action potential for five amplitude levels in descending order downwards, and the lower traces show the analog signals of the corresponding stimuli.

The wave of mechanical indentation for SA1, RA1, and PC units were set referring to the literature of Mountcastle et al. (1966), Talbot et al. (1968) and Knibestöl (1973), respectively. As seen in Figure 4, SA1 units respond to the onset and hold phase but typically not its offset stage. The firing rates in SA1 afferents increase almost linearly as indentation depth increases. The firing rate is higher during the dynamic phase of indentation than during steady indentation. When the probe is removed from the skin, the spike activity ceases. RA1 units respond strongly to onset and offset but typically adapt so quickly that they do not respond during static indentation. RA1 afferents can signal the rate at which the stimulus is applied and removed: rapid changes evoke a brief burst of high-frequency spikes, whereas slow changes evoke a longer-lasting, low-frequency spike train. PC receptor is highly sensitive to acceleration and higher derivatives. The PC afferents respond not only when the indentation is increasing, but also when the stimulus is retracted, however they are not very sensitive to punctate stimuli and completely independent of indentation velocity and amplitude. The above simulated responses of the three afferent types match well with the neurophysiological observed counterparts.

#### Frequency Characteristics of Neural Threshold

**Figure 5:**
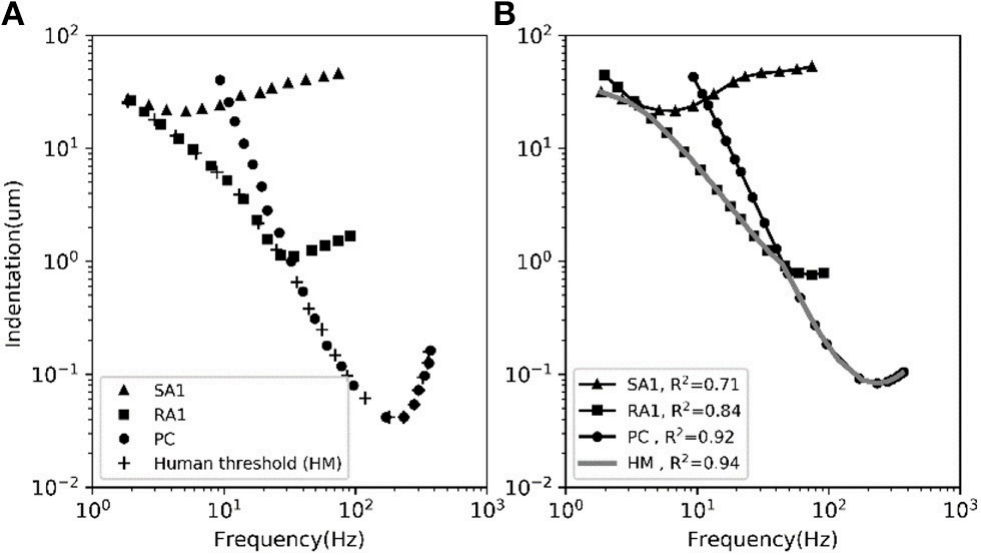
The average frequency-threshold curves of single-unit. **(A)** Observations [adapted from SA1, RA1 (Verrillo and Bolanowski Jr, 1986), PC (Mountcastle et al., 1972), HM (Gescheider et al., 1985)]. **(B)** Prediction of our model. The human threshold (HM) is the minimum of the thresholds of the 3 mechanoreceptive types. The R2 in the legend is the coefficient of determination between the observed data in panel **(A)** and the predicted data in panel **(B)** for each afferent and for the human threshold (gray).

The frequency sensitivity for each type of tactile unit is reflected in its frequency characteristics of neural threshold as illustrated in Figure 5A. In current model, since the firing rate of the outputting spikes is determined by the output signal of *v*_nf_, the predicted frequency characteristics of neural threshold for each afferent type can been a calculated according to equation (3). As shown in Figure 5B, the human thresholds for vibration closely match those of the most sensitive afferent fibers in each range (*R*^2^ = 0.94).

### Reproducing Spiking Timing

As indicated in neurophysiological experiments investigating tactile encoding in the nerve, the cutaneous tactile afferents exhibit very precise and repeatable timed spike responses to vibratory stimuli (Talbot et al., 1968). The importance of spike timing in tactile coding has since been established across a variety of tactile sensory modalities (Saal et al., 2016), including vibratory frequency (Harvey et al., 2013), surface texture (Weber et al., 2013), surface curvature (Mackevicius et al., 2012), and direction of tangentially applied forces (Johansson and Birznieks, 2004). To evaluate the performance of this model in reproducing spike timing, we carried out another simulation by using the recorded data of spiking responses to sinusoidal vibration from Muniak et al. (2007).

**Figure 6:**
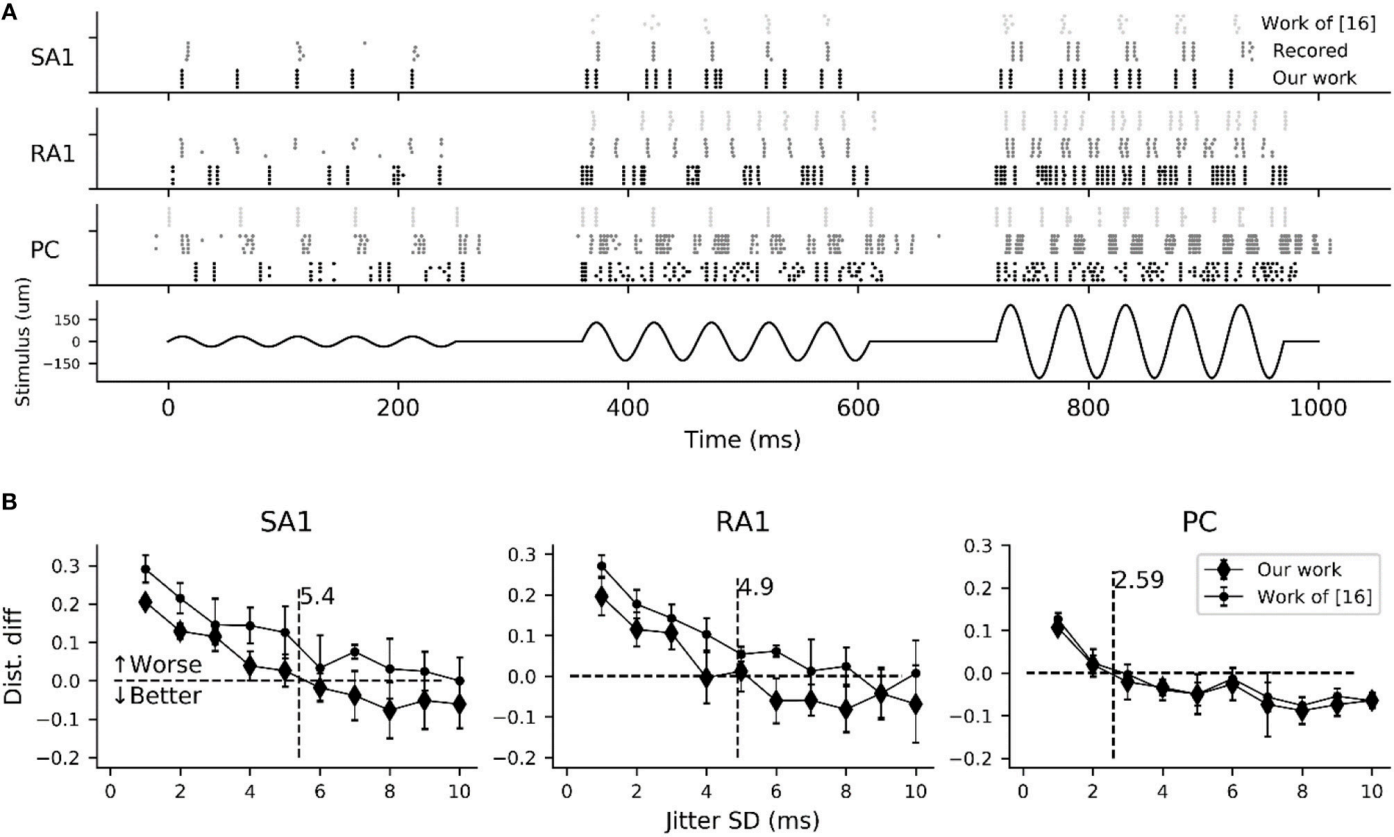
Evaluation of spike-timing precision for each afferent type. **(A)** Recorded (gray dotted marks) and simulated spike trains of 5 sampled tactile units for each afferent type in response to a sinusoidal stimulus of 20 Hz vibration (black solid line) with amplitudes of 35 (left), 130 (center) and 250 um (right). Recorded spike trains and the stimulus were adapted from Muniak et al. (2007). **(B)** Difference in the spike distance between the simulated and jittered spike trains to recorded spike trains as a function of jitter SD. The vertical black dotted line localizing at the cross of zero horizontal line and the quadratic fitting curve of current model determine the timing precision.

As shown in Figure 6A, the simulated responses to sinusoidal stimuli match well with their recorded counterparts. To quantify the timing precision of current model in predicting the recorded spiking responses, we computed the similarity between the simulated and recorded spike trains at different time scales using the metric of ISI (interspike interval) distance (Kreuz et al., 2007). We could then determine how much we needed to jitter the recorded spike trains to achieve the level of temporal precision of the simulated responses using distance difference (dist. diff, referring to Saal et al., 2017). The jittered spike trains was generated by sampling randomly from a zero-mean Gaussian distribution with a given SD and then adding to each recorded spike (Bensmaia et al., 2008). We tested SDs ranging from 1 to 10 ms. As illustrated in Figure 6B, the model is worse if the averaged difference is greater than zero (horizontal black dotted line). The jitter value that averaged difference curve cross zero was defined as the precision of the model (vertical black dotted line Figure 6B). We found that the current models of all afferent types achieve a temporal precision better than 6 ms. The PC models are the most precise, down to precision of about 2.5 ms, The SA1 and RA1 models achieve precisions ranging from 3 to 6 ms.

In order to compare current model with the work (Saal et al., 2017) in terms of timing precision, we also reproducing the spiking trains in response to the same stimulus using code in work of (Saal et al., 2017) (http://bensmaialab.org/code/touchsim/). The code of reproducing the spike trains as shown in Figure 6B are also available in our Github repository. As shown in Figure 6B, the current model performs relatively better the work of (Saal et al., 2017), especially for SA1 afferents.

## Performance Evaluation and Comparison With Previous Work

### Computational Efficiency

**Figure 7:**
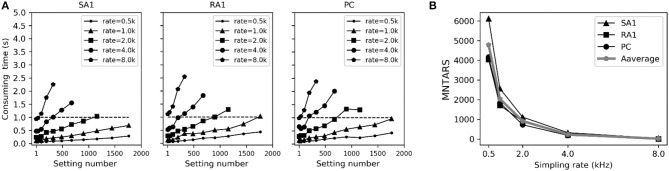
Evaluation of computation efficiency for simulating population units. **(A)** Averaged consuming time for simulating each type of tactile unit for 1 second at different setting number under different sampling rate. Error bar represent the standard deviation of the mean. **(B)** MNTARSs at different sampling rates. The MNTARS was evaluated as the setting number at cross of consuming time curve and horizontal dotted line in panel **(A)**.

To evaluate the computational efficiency of simulating massive numbers of tactile units, we present the averaged consuming time and the maximum number of tactile units that allow real-time simulation (MNTARS) at different sampling rates for each afferent type (Figure 7). When simulating multiple tactile units, the input, all intermediate or output variables were written in a two-dimensional Numpy arrays (see Figure 1D), and the consuming time was measured with time stamp of Python. As seen in Figure 7B, the model runs in real time with about 300 units at a sampling rate of 4 kHz, and 2,000 units at rate of 1 kHz, and 4,000 units at rate of 500 Hz (running under the condition of 62% remaining free physical memory).

### Comparisons With Previous Work

In order to compare with the previous spike neuron models, we also implemented the Hodgkin-Huxley, FitzHugh-Nagumo, Izhikevich Model, and IF models referred from the literature (Hodgkin and Huxley, 1952; Fitzhugh, 1961; Izhikevich, 2003; Brette and Gerstner, 2005), respectively. The codes of these compared models were rewritten with Python and available in our Github repository. The NSM model was not implemented for comparison, since its code is not available.

**Figure 8:**
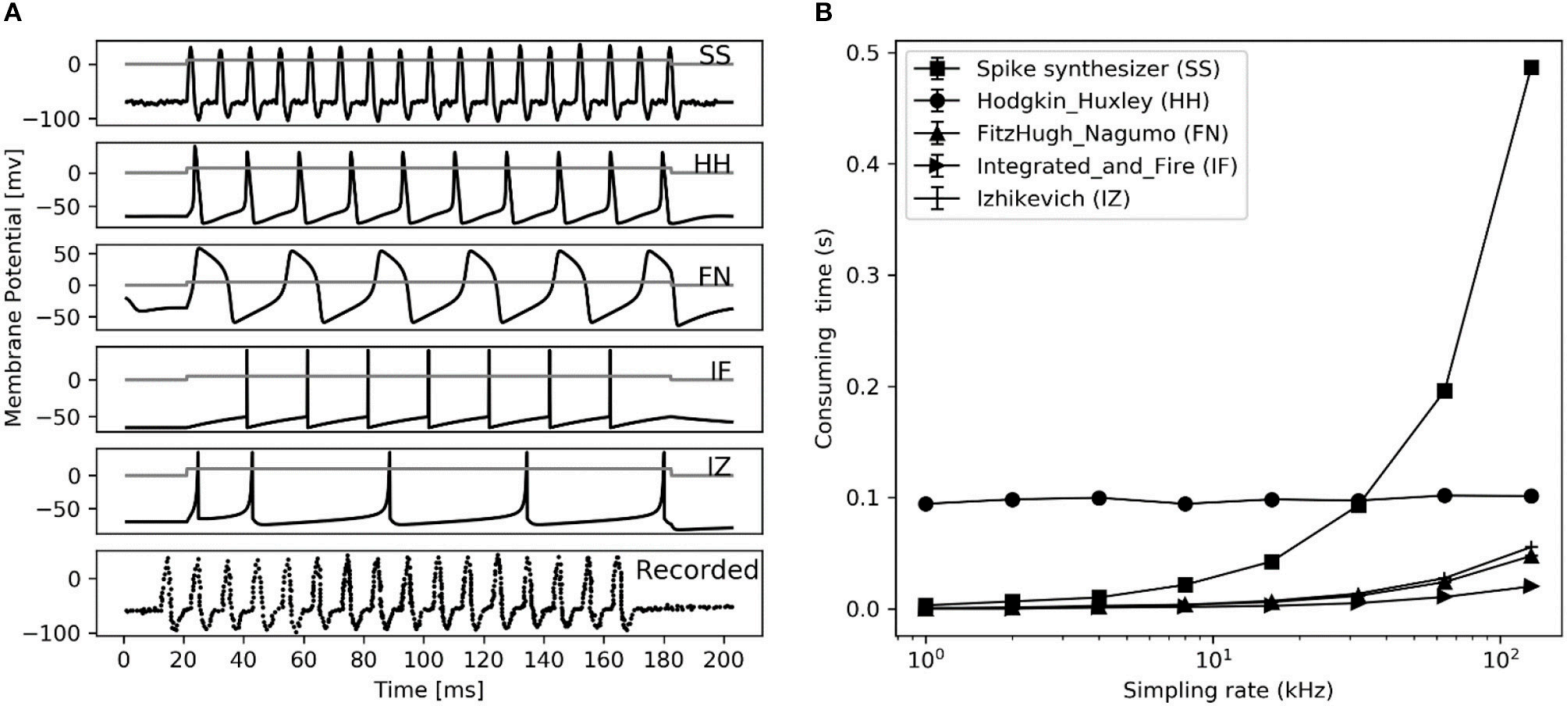
Performance comparison between different spiking neuron models. **(A)** Reproduction of spiking waveform (200 ms frame) using our spike synthesizer and previous neuron models (from top 1 to 5th trace), their applied current (*i*_a_, nA) is shown in gray curve in each simulated trace. The recorded typical spiking waveform reprinted from Hodgkin et al. (1952), are depicted in bottom. **(B)** Consuming time of converting applied current into action potential for simulation in left figure at different sampling rate using different neuron models. Error bars represent the standard deviation.

Comparing firing spike reproduction by the previous spiking neuron models, the spike waveform generated by spike synthesizer matches the typical recorded ones almost perfectly (see Figure 8A). Although the spike synthesizer did not show computational efficiency over some spiking neuron models (e.g., FN, IF, IZ, see Figure 8B) especially at high sampling rate, improvement of matching recorded spiking shape (6th trace in Figure 8A) over these models is dramatic. In addition, the spike synthesizer can be implemented to accurately reproduce action potential of any shape by changing base wave in Figure 1B.

A multifaceted comparisons between this model and previous related work are shown in Table 2. Compared to the NSM model and the Work of (Saal et al., 2017), our model have less parameters, but it can be implemented to characterize 3 major types of tactile units, and allow for a faster computation of reproducing most of their response properties with higher timing precision.

**Table 2:** A comparison between the contributions of this work and the related works.

**Models**	**Parameter number**	**MNTARS (units)**	**TP (ms)**	**Properties allowed to be simulated**			**Type of afferent fiber**		
				**FC**	**AT**	**AP**	**SA1**	**RA1**	**PC**
This work	10	≈4000	<6	Y	Y	Y	Y	Y	Y
Work of Saal et al., 2017	13	<350	<7	Y	Y		Y	Y	Y
NSM model Biswas et al., 2015	>20			Y	Y	Y	N	N	Y

*TP, Timing precision; FC, Frequency sensitivity; AT, Absolute threshold; AP, adaptation properties; Y, supported; N, unsupported*.

## Discussions

In the present work, we simulated the responses of single tactile receptors and their afferents activated by a punctate or vibrating stimuli. The current model creates a biologically plausible response using many characteristics taken from the known neurophysiological literature.

As depicted in Figure 5B, the adaptation properties of simulated units (Figure 4) create accurate frequency characteristics of neural threshold (Figure 5B). It supports the hypothesis that frequency characteristics of neural threshold is mostly due to the adaptation properties (Bolanowski et al., 1988). As illustrated in Figure 6, we found a high temporal agreement between the observed spike timing and that predicted by this model, which can be attributed to the training method using neurophysiological dataset. Compared to model developed by Saal et al. (2017), this model make use of frequency modulator as illustrated in Figure 1B to generate action potentials whose firing rate is linear to the output of TCF model. Therefore, the current model can be simplified easily and trained accurately, which allows for quickly simulating thousands of tactile units with high timing precision (see Figures 6, 7B).

The model developed by Saal et al. can also be implemented to reproduce spiking responses with millisecond precision for the 3 types of tactile units, and runs in real time with 300 units at a rate of 300 Hz (Saal et al., 2017). Their work includes an IF model, and the continuum mechanics (CM) model to simulate the skin mechanics. As illustrated in Figure 6, the current model achieve almost same timing precision for PC units, but performs better for SA1 and RA1 units. As seen in Figure 7B and Figure 8B, the current model allow for the real-time simulation of about 4,000 tactile units at a rate of 500 Hz and reproducing the accurate spike shape of tactile afferents. The spiking synthesizer can be implemented to reproduce biological action potentials of different tactile neuron types by replacing different base waves (see Figure 1C). Since there is a diversity in shape of action potentials in peripheral and central neurons, the spiking synthesizer may be extended simulate other sensory neurons (Bean, 2007). Compared to model developed by Saal et al. the current work lack of CM model to simulate skin mechanics. The CM model adopted by Saal et al is fast to compute (Sripati et al., 2006), thus the current model combining with the CM model will perform better in biological compatibility and computational efficiency than the model by Saal et al.

It should be noted that the simulations were mostly compared to responses recorded from nerves innervating the monkey fingertip, but the model can also be expanded for the human by only adjusting the mechanotransduction coefficient (*A*_s_), since the individual tactile afferents in human and macaque behave nearly identically in basic response properties (Muniak et al., 2007). Interestingly, we also found that all three types of mechanoreceptors were sensitive to a stimulus with a sudden ramp change. It is common among sensory systems for dynamic stimuli to generate short, but strong, responses. This can be seen in vision (Bisley et al., 2004), and the auditory system (Flint et al., 2011), as well as in the tactile system. This phenomenon could be associated with orienting reflex, which is an organism's immediate response to a change in its environment. Animals constantly focus attention to changes of the stimulus, which is an important way to survive.

## Applications

A mathematical model to quantitatively characterize tactile unit in response to stimuli and to output a response in a biological code has great potential. For instance, the proposed model can be a powerful tool to investigate the sense of touch. Indeed, recording the responses of human or monkey afferents is technically challenging and only yields responses at a time. The model could also be used to create hypotheses about how more complex stimuli are encoded in the tactile periphery, which can then be tested in the human or animal model. The whole hand contains about 17,000 tactile units (Johansson and Vallbo, 1979), which work together to transmit information about shape, texture and movement. After a certain simplification, our model allows for real-time simulating the responses of tactile afferents in whole hand.

The model can be used as a tool to investigate shape and texture encoding in nerve system. The PC afferents play a role in the perception of fine textures whose elements are too small and closely spaced to be processed spatially (Hollins et al., 2002), and even for the perception of relatively coarse textures (Cascio and Sathian, 2001; Gamzu and Ahissar, 2001). This model lays the groundwork for researchers to investigate the interactive effect between PC and SA1 afferent responses to fine textures, which provide a valuable insights to improving realism of rendering virtual fine texture with haptic devices.

An exciting use of this model is in brain-machine interfaces. Using a model such as this, signals transduced by sensors located on a prosthesis could be converted into patterns of neural activity, which could then be sent to a peripheral nerve to accurately represent how the nerve in hand would respond to skin indentation. In addition, the spiking synthesizer could be used to convert any spike rate input into a burst of biological action potentials with high timing precision, which may improve the biocompatibility of the current sensory prosthesis. The current model may also leads to new ways of designing highly realistic tactile interfaces such as neurorobots and bionic hands (Bologna et al., 2001).

## Limitations of Model

This model faithfully reproduces key response properties of tactile afferents, but it is also subject to some limitations.

First, while we were able to simulate single-afferent response for SA1, RA, and PC afferents, we did not simulate SA2 fibers, since there are very few neurophysiological experimental data for fitting the SA2 model, and the primary function of SA2 fibers is thought to be encoding stretch, rather than indentation (Johnson, 2001).

Second, this model treats the tactile unit as a single-unit isolated from other units, when in reality, it is not. Histological studies reveal that the connections are ubiquitous in tactile afferents, with single afferent fibers innervating multiple receptors and single receptors receiving multiple afferent fibers (Par et al., 2002). However, the model can be extended to accommodate afferent branching, especially when mechanisms by which input from the multiple receptors is integrated to evoke the afferent responses are better understood by us Lesniak et al. (2014).

Third, the current model rapidly recreates the temporal firing characteristics of tactile units with high temporal precision, however the mimicry of their spatial characteristics is not addressed. The spatial characteristics of spikes is reflected in afferent population response (Yoshioka et al., 2001; Weber et al., 2013). To simulate population response, parameters such as the skin elasticity and relative positions between the contact points and the receptors on the contact area are required. The skin biomechanics which includes these parameters can be included in has been proven to be important to simulating tactile afferent response (Birznieks et al., 2010). Although skin mechanics is not considered in this study, the current model is also useful. As shown in Figure 1D, all the signal variables were defined as two dimensional arrays to set an interface for simulating population responses. By adding an accurate skin mechanics model to compute the skin indentations from stimuli, the current model has the capability of reproducing afferent population response to stimuli of different shape and size with high timing precision.

## Conclusion

The current work provides a pieces of Python code to accurately reproduce the selective responses of single tactile units in a biological pattern using an electromechanical circuit model. This model has the potential of giving us a better understanding of afferent firing patterns in response to skin indentations. This work may provide valuable guidance to designing prostheses with tactile feedback, enhancing the realism of haptic rendering of virtual tactile stimuli, and building a digitized human hand with physiological response in a virtual surgical system.

Although the current work has mimicked the responses of single tactile receptors and afferents comprehensively, it is not enough to recreate the tactile afferent population responses. Complex tactile stimuli such as texture, shape, roughness of a grating, and edge orientation, are encoded in population responses (Khalsa et al., 1998; Hollins and Bensmaïa, 2007; Weber et al., 2013; Suresh et al., 2016). In the future, we will address these issues by constructing a computing model of population responses. This is beyond the scope of the current work but we think this might allow for a better understanding of peripheral representation of tactile stimuli.

## Author Contributions

QO and JW designed the simulation code. QO implemented the system, analyzed the data, and wrote the article. JW reviewed the paper and contributed to the writing and to the data analysis. ZS, MW, and ZC contributed to the collecting data from previous neurophysiological literature. ZS, MW, and ZC contributed to the English usage proofing.

### Conflict of Interest Statement

The authors declare that the research was conducted in the absence of any commercial or financial relationships that could be construed as a potential conflict of interest.
